# 

**Published:** 1875-08

**Authors:** 


### CONTENTS:

ORIGINAL COMMUNICATIONS:
ART. I. Two Cases of Aphasia. By Walter Hay, M.D., Chicago ........561
ART. II. The Cheaper Cinchona Alkaloids. By Jas. S. Whitmire, M.D., Metamora, Ill. ......................564
ART. III. Successful Operation for Stone in the Bladder. By E. O. Gratton, M.D., Hebron, Ill. .........569

PROGRESS IN MEDICAL SCIENCES:
ART. I. Progress of Surgery. By Jno. E. Owens, M.D., Chicago. ..... . ..570

REPORTS OF SOCIETIES:
Chic. Society Physicians and Surgeons, 575
Chicago Medical Society .. ..........577
Wisconsin Medical Society ............587
Cass Co. (Ind.) Medical Society .......596
Am. Neurological Association .........598

SELECTIONS ...........................602
EDITORS' BOOK TABLE..... .. .... ....606
MEDICAL ITEMS, NEWS AND GOSSIP ....617
EDITORIAL .................... ........623
LOOT .... ...........................627
MORTALITY STATISTICS, Chicago.........639

